# L-cysteine modulates visceral nociception mediated by the Ca_V_2.3 R-type calcium channels

**DOI:** 10.1007/s00424-022-02674-y

**Published:** 2022-03-10

**Authors:** Seyed Mohammadreza Ghodsi, MacKenzie Walz, Toni Schneider, Slobodan M. Todorovic

**Affiliations:** 1 Department of Anesthesiology, University of Colorado, CO Aurora, USA; 2 Neuroscience Graduate Program University of Colorado Anschutz Medical Campus, Aurora, CO 80045 USA

**Keywords:** R-type channels, Hyperalgesia, Inflammatory pain, Acetic acid, Visceral nociception

## Abstract

Ca_V_2.3 channels are subthreshold voltage-gated calcium channels that play crucial roles in neurotransmitter release and regulation of membrane excitability, yet modulation of these channels with endogenous molecules and their role in pain processing is not well studied. Here, we hypothesized that an endogenous amino acid l-cysteine could be a modulator of these channels and may affect pain processing in mice. To test this hypothesis, we employed conventional patch-clamp technique in the whole-cell configuration using recombinant Ca_V_2.3 subunit stably expressed in human embryonic kidney (HEK-293) cells. We found in our in vitro experiments that l-cysteine facilitated gating and increased the amplitudes of recombinant Ca_V_2.3 currents likely by chelating trace metals that tonically inhibit the channel. In addition, we took advantage of mouse genetics in vivo using the acetic acid visceral pain model that was performed on wildtype and homozygous *Cacna1e* knockout male littermates. In ensuing in vivo experiments, we found that l-cysteine administered both subcutaneously and intraperitoneally evoked more prominent pain responses in the wildtype mice, while the effect was completely abolished in knockout mice. Conversely, intrathecal administration of l-cysteine lowered visceral pain response in the wildtype mice, and again the effect was completely abolished in the knockout mice. Our study strongly suggests that l-cysteine-mediated modulation of Ca_V_2.3 channels plays an important role in visceral pain processing. Furthermore, our data are consistent with the contrasting roles of Ca_V_2.3 channels in mediating visceral nociception in the peripheral and central pain pathways.

## Introduction

Voltage-gated calcium channels (VGCCs) play essential roles in the nervous system including gene expression (L-type), neurotransmitter release (N-, P/Q-, and R-type), and membrane depolarization (T-type). The family of VGCCs consists of the pore-forming α1 subunit that provides the binding sites for agonists and antagonists. The Ca_v_2.3 (former α1E-subunit) or R-type channel is a subtype of the VGCC family that was discovered and cloned relatively late, partly due to its resistance to the conventional calcium channel antagonists [16, 22]. Subsequent studies have shown that neuronal Ca_v_2.3 channels in the periphery, spinothalamic, and thalamocortical tracts contribute to gene translation and cell membrane excitability [18]. Immunohistochemistry experiments have shown the abundance of Ca_V_2.3 protein in the dorsal root ganglia (DRG) and reticular nucleus of thalamus (RTN), as well as neocortex, suggesting their possible role in peripheral and central nociception and underlying sensory processing [5, 18, 23].

The role of endogenous redox and chelating agents, including amino acids and peptides in regulating pain processing, has been a sought-after subject in pain research. Electrophysiological studies have reported enhanced firing frequency in neurons in the presence of endogenous reducing amino acid l-cysteine [7]. L-cysteine could be found locally in the interstitial spaces in relatively high concentrations under conditions of metabolic stress or tissue injury [11]. Although previous studies have suggested a few specific pathways for the action of these substances, the thorough mechanism remains elusive and sometimes controversial. For example, l-cysteine in the extracellular environment may oxidize cysteine groups on the ion channel membrane thereby manipulating channels’ natural dynamics [4, 21]. On the other hand, there are arguments on the chelating properties of l-cysteine rather than oxidizing, where chelating agents regulate trace metals in the environment thus manipulating function of an ion channel [8].

Our group previously reported that Ca_V_2.3 channel function in sensory neurons can be inhibited by trace metals and facilitated with certain endogenous chelating agents such as glutamate [19]. However, functional significance of such modulation has not been previously studied. Hence, we hypothesized that modulation of the Ca_V_2.3 channels via endogenous redox/chelating agents could affect pain processing in mice. Herein, using human recombinant Ca_V_2.3 subunit stably expressed in human embryonic kidney cells (HEK-293) and mouse genetics, we have studied the role of Ca_v_2.3 channels and an endogenous amino acid such as l-cysteine on sensitization of visceral pain responses.

## Materials and methods

### Human embryonic kidney 293 cells

Human variant of Ca_V_2.3 was stably transfected in HEK-293 cells that were grown in DMEM (Sigma-Aldrich D5796-6X500ML) supplemented with 10% fetal calf serum (Sigma-Aldrich F0679-500ML), 100 U/ml penicillin (Sigma-Aldrich P4333-100ML), 0.1 mg/ml streptomycin (Sigma-Aldrich P4333-100ML), and 0.5 mg/ml gentamycin (Sigma-Aldrich A1720-1G), and 0.2 mg/ml hygromycin-B to stabilize channel expression (Sigma-Aldrich H3274-250MG) [18].

### Electrophysiology

Electrophysiological experiments were performed using the conventional patch-clamp technique in the whole-cell configuration at room temperature (22 °C) using Cornerstone PC-ONE amplifier and Digidata 1322A with pClamp 10.7 software (Molecular Devices). Data were filtered at 2 kHz and digitized at 5 kHz. Cell capacitance, membrane resistance, access resistance, and holding potential were measured using the built-in Membrane Test function. The average access resistance was around 4 MΩ. Currents were recorded in the following external solutions (in mM, all purchased from Sigma-Aldrich): 160 TEA-Cl, 10 BaCl_2_, 0.1 EGTA, and 10 HEPES, pH adjusted to 7.4 with TEA-OH. Pipettes for recordings (around 4 MΩ resistance) were filled with internal solution of the following composition (in mM): 100 Cs-methane sulfonate, 14 phosphocreatine, 10 HEPES, 9 EGTA, 5 Mg-ATP, 0.3 Tris-GTP, pH adjusted to 7.3 with Cs-OH. The recording chamber was constantly perfused with the extracellular solution at 1 ml/min.

### Animals

Experimental procedures with animals were performed according to a protocol approved by the Institutional Animal Care and Use Committee of the University of Colorado Anschutz Medical Campus, Aurora, CO, USA. Treatments of animals adhered to guidelines set forth in the NIH Guide for the Care and Use of Laboratory Animals. All efforts were made to minimize animal suffering and to use only the necessary number of animals to produce reliable scientific data. Wild-type and homozygous *Cacna1e* mutant male C57BL/6 J littermates were obtained from the Mutant Mouse Resource and Research Centers (MMRRC) and used for pain behavior studies as outlined elsewhere [15]. All animals were maintained on a 14-/10-h light/dark cycle with food and water ad libitum. Behavioral assessments for in vivo experiments were done in a blinded fashion.

### Acetic acid visceral pain model

Visceral pain was induced by injection of 300 µl acetic acid (in 0.6% saline) intraperitoneally (i.p.). L-cysteine was administered either systemically at10 mg/kg subcutaneously (s.c.), or 36 µg/300 µL intraperitoneally (i.p), or intrathecally. For intrathecal (i.t*.*) administrations, animals were anesthetized with 2% isoflurane, and 1.2 µg/10 µl l-cysteine solution in saline was injected between L4 and L5 vertebrae about 20 min prior to i.p. acetic acid injections. The correct i.t. injection site was confirmed by observing subjects’ tail flick. Writhing reflex of subjects were recorded and scored for 30 min post acetic acid injections [2].

### Analysis

Data were analyzed using Clampfit 10.7 (Molecular Devices), MS Excel 2007 (Microsoft), and GraphPad Prism 9. Smooth curves in the figures represent fits to the average data, whereas the mean values in the table were calculated from fits to individual cells. Data are reported as the mean ± SEM. We used either one-way ANOVA, to compare multiple mean values, or a paired Student’s *t*-test when evaluating the effect of acute application of agents. The voltage dependence of activation and inactivation was determined by fitting the peak current–voltage (I–V) data from each cell with a Boltzmann-Goldman–Hodgkin–Katz (GHK) equation $G(V) = {G}_{\mathrm{max}}/1+\mathrm{exp }[-(\mathrm{V}-{V}_{50})/k]$ and $\mathrm{I}\left(\mathrm{V}\right)=\frac{{I}_{\mathrm{max}}}{1}+\mathrm{exp }\left[-\frac{V-{V}_{50}}{k}\right].$

## Results

### L-cysteine augments calcium currents in Ca_v_2.3-transfected HEK-293 cells at physiologically relevant concentrations

**Fig. 1:**
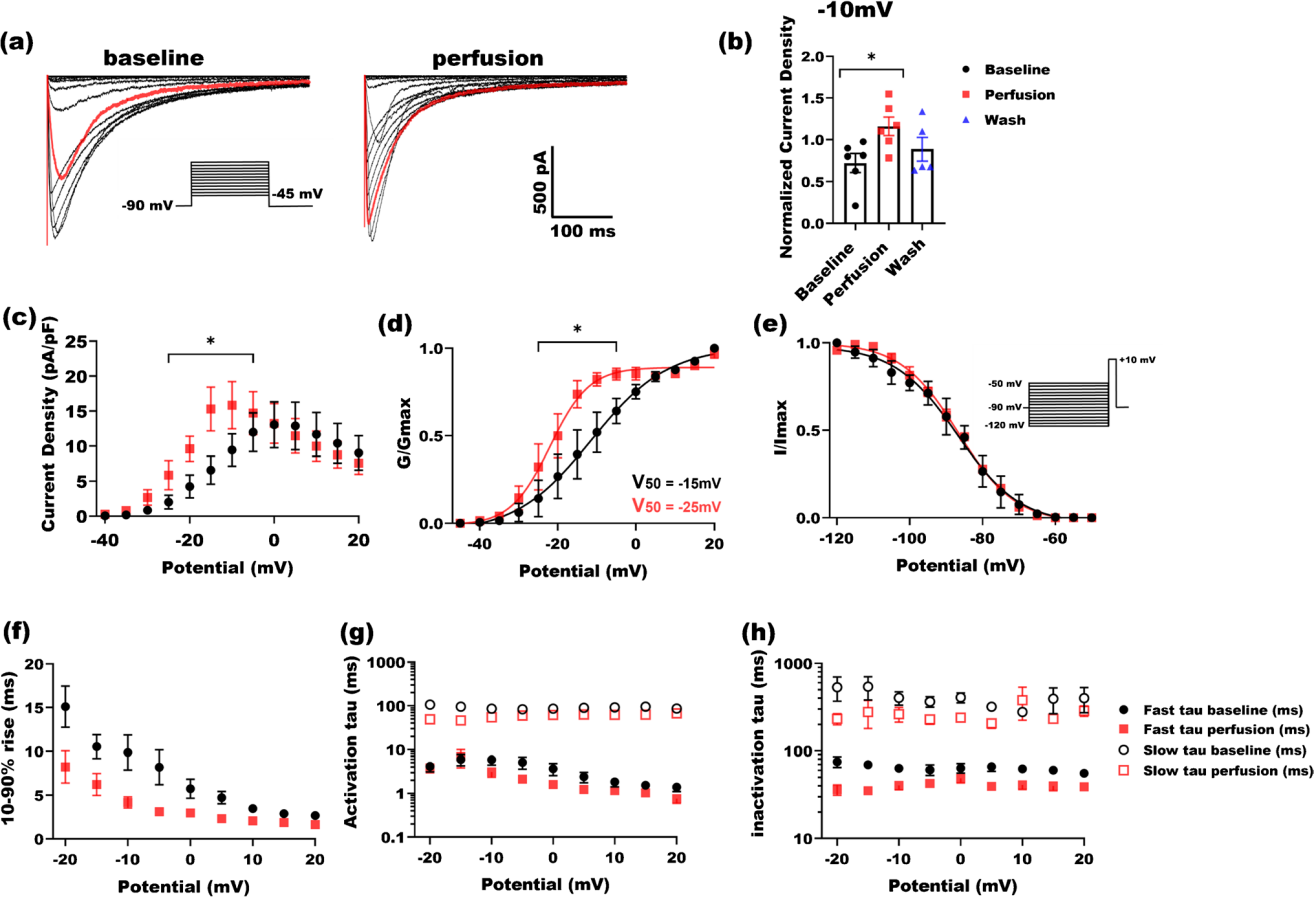
The effect of 50 µM l-cysteine on the kinetic properties of Ca_v_2.3 currents. **a** Traces of inward barium currents arising from R-type calcium channels in Ca_v_2.3-transfected HEK-293 cells. Perfusing cells with 50 µM l-cysteine solution in TEA increased currents amplitude significantly from the baseline. Red traces depict the current at − 10 mV test potential in either baseline and perfusion (inset: I–V protocol). **b **Normalized current densities at − 10 mV are shown for 6 cells. Perfusing cells with 50 µM l-cysteine increases Ca_V_2.3 currents significantly (paired two-tailed *t*-test, *P* = 0.03). All currents are normalized to the maximal current density in baseline conditions. **c** Current densities across different test potentials from 6 cells are drawn in the presence (red) and absence (black) of 50 µM l-cysteine. A significant increase of current was observed upon perfusion at − 20 to − 10 mV test potentials (two-way ANOVA *P* = 0.01 with Sidak multiple comparisons). **d** Normalized conductance of Ca_V_2.3 channels across test potentials is derived and sketched to compare the activation kinetics of Ca_v_2.3 channels with and without 50 µM l-cysteine, where a significant increase was observed at − 25 to − 5 mV potentials (two-way ANOVA *P* = 0.01 with Sidak multiple comparisons). **e** Inactivation protocol (inset) did not reveal any significant difference between inactivation kinetics after perfusing cells with 50 µM l-cysteine. **f** Next, 10–90% rise time was derived from Ca_V_2.3 current traces at different potentials. Overall 10–90% rise time for 50 µM l-cysteine was significantly faster than the baseline (two-way ANOVA *P* = 0.03 with Sidak multiple comparisons). **g** Fast and slow time constants of activation were derived by fitting two-exponential function to Ca_V_2.3 current waveforms. **h** Fast and slow time constants of inactivation were derived by fitting two-exponential function to Ca_V_2.3 current waveforms

HEK-293 cells were perfused with external solution alone (baseline) and 50 µM l-cysteine in external solution (perfusion) consecutively to study the effect of l-cysteine on the gating of Ca_v_2.3 channels (Fig. 1). Current densities were obtained by normalizing the peak current amplitude of each cell to its capacitance and averaging across all examined cells and plotted against test potentials accordingly. Figure 1a depicts inward traces for the baseline and perfusion, where red traces are recorded at − 10 mV test potential in either experiment. We noted that 50 µM l-cysteine robustly increased barium current from the baseline about twofold (peaking at -10 mV) with only partial recovery in most of the tested cells. Peak current densities at − 10 mV test potential are shown in Fig. 1b. Figure 1c shows the I–V curve for the baseline (black circles) and perfusion (red squares) where 50 µM l-cysteine has significantly shifted the peak to hyperpolarized potentials by about 5 mV. To accurately determine the R-type calcium channels gating properties in the presence of 50 µM l-cysteine, activation and inactivation protocols were applied (Fig. 1d, e). While no significant difference was observed in the inactivation kinetics between two groups, l-cysteine shifted V_50_ of activation to hyperpolarized potentials by about 10 mV. Analyzing 10–90% rise time in I–V curves in Fig. 1f revealed that l-cysteine significantly speeds up Ca_v_2.3 currents compared to the baseline. We fitted and analyzed activation and inactivation curves (Fig. 1g, h respectively) for each cell and averaged among the test potentials to obtain fast and slow time constants. Overall, Fig. 1 proved the significant effect of 50 µM l-cysteine on facilitation of channel gating and augmenting R-type calcium current densities. In order to investigate the interaction of l-cysteine with Ca_v_2.3 channels under higher pathologically relevant concentrations (e.g., inflammation), we repeated our experiment using 500 µM l-cysteine.

### L-cysteine augments R-type calcium currents at high concentrations

**Fig. 2:**
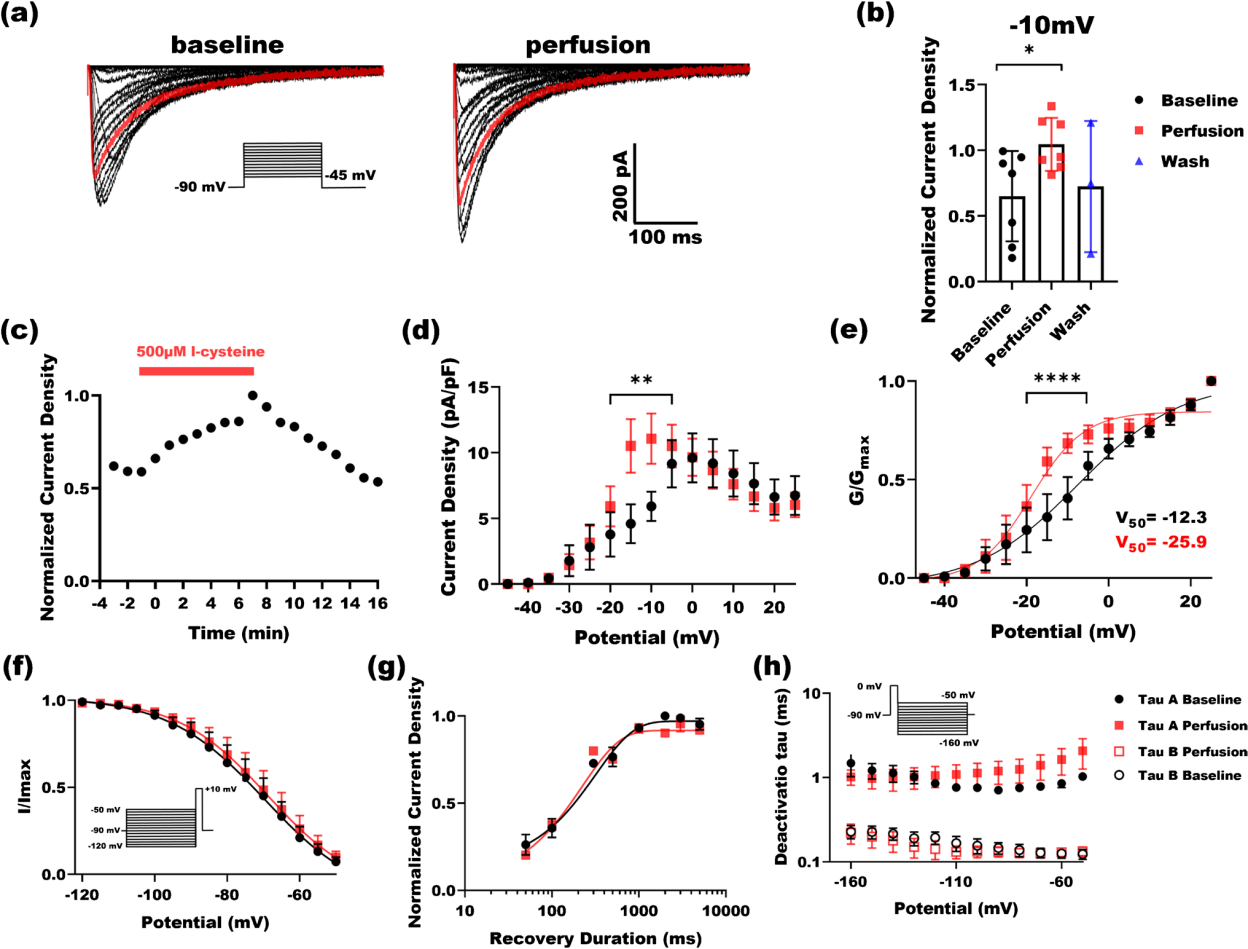
The effect of 500 µM l-cysteine on the kinetic properties of Ca_v_2.3 currents. **a** Traces of inward barium currents arising from Ca_v_2.3 calcium channels in stably transfected HEK-293 cells. Red traces are recorded at − 10 mV test potential. Note that perfusing cells with 500 µM l-cysteine solution significantly increased currents when compared to the baseline (inset: I–V protocol). **b** Normalized current densities at − 10 mV are shown for 7 cells. Perfusing cells with 500 µM l-cysteine increases currents significantly at − 10 mV test potentials (paired two-tailed *t*-test, *P* = 0.01). All currents are normalized to the maximal current density in baseline conditions. **c** A representative time course of effect of 500 µM l-cysteine perfusion on Ca_V_2.3 current over 6 min. Washing with external solution brought the current back to baseline within 9 min. **d** Current density from 7 cells are drawn against test potentials in the presence (red) and absence (black) of 500 µM l-cysteine. A significant increase was observed upon perfusion at − 20 to − 5 mV test potentials (two-way ANOVA *P* = 0.004 with Sidak multiple comparisons). **e** Normalized conductance of Ca_V_2.3 channels across test potentials are derived and depicted to compare the activation kinetics of Ca_v_2.3 channels w/o 500 µM l-cysteine, where a significant increase was observed at − 20 to − 5 mV potentials (two-way ANOVA *P* < 0.0001 with Sidak multiple comparisons). **f** Inactivation protocol (inset) did not reveal any significant difference between inactivation kinetics after perfusing cells with 500 µM l-cysteine. **g** Recovery from inactivation duration protocol was ran with and without 500 µM l-cysteine and plotted against the normalized current density; no significant difference was observed between baseline and perfusion. **h** Deactivation protocol (inset) was applied on cells in the presence and absence of 500 µM l-cysteine and fitted with two-exponential function to derive fast and slow deactivation time constants, then plotted against the test potentials, no significant difference was observed among fast or slow tau

While 50 µM l-cysteine proved to be an effective endogenous R-type calcium current modulator at low concentrations, the question about the role of pathologically relevant higher doses of l-cysteine that may exist under pain or inflammation remains unclear. Figure 2 summarizes patch-clamp electrophysiological recordings in Ca_v_2.3-transfected HEK-293 cells perfused with 500 µM l-cysteine. Figure 2a depicts barium current traces for baseline and perfusion. Red traces are recorded at − 10 mV test potential where perfusion has increased the inward current compared to the baseline. Similar to lower concentration, we found that at 500 µM l-cysteine significantly increased current from the baseline about twofold (Fig. 2a, b) peaking at − 10 mV. In Fig. 2c, we show that 500 µM l-cysteine was applied and washed in the course of 16 min to find out that it reversibly increased R-current amplitude with a relatively fast on and off kinetics. Figure 2d shows the I–V curve for the baseline (black circles) and perfusion (red squares) where 500 µM l-cysteine has significantly shifted the peak to hyperpolarized potentials by about 10 mV. The Ca_V_2.3 channel gating in the presence of 500 µM l-cysteine was determined by running activation (Fig. 2e) and inactivation (Fig. 2f) protocols. Similar to 50 µM concentration, 500 µM l-cysteine perfusion left inactivation kinetics of Ca_v_2.3 channels practically intact; however, V_50_ of activation was shifted to hyperpolarized potentials by over − 13 mV. Figure 2g and 2h investigate the possible role of 500 µM l-cysteine in recovery from inactivation and deactivation time constants of Ca_V_2.3 channels; however, no significant effect was observed.

### L-cystine blocks Ca_V_2.3 currents

**Fig. 3:**
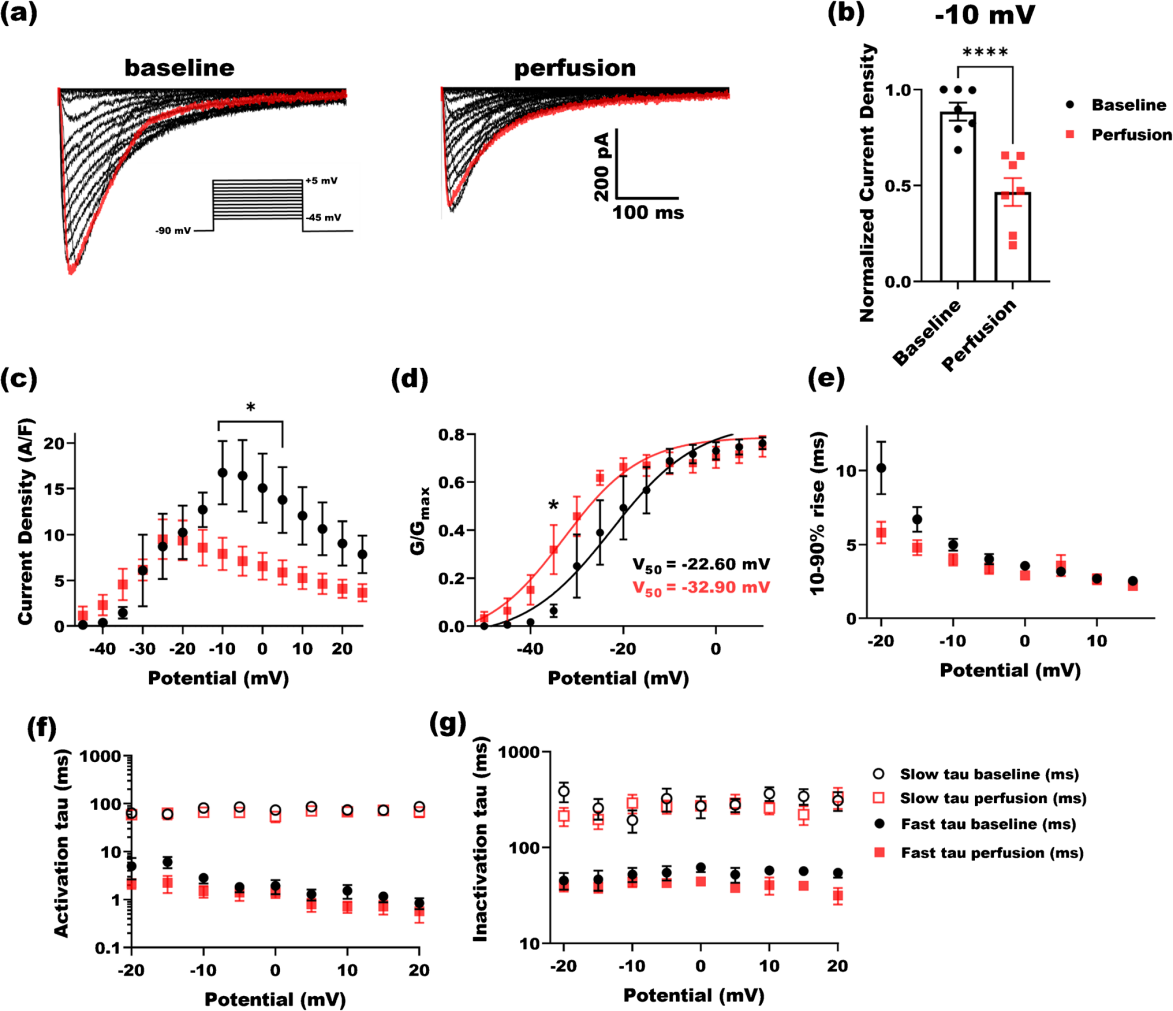
The effect of 500 µM l-cystine on the kinetic properties of Ca_v_2.3 currents. **a** Traces of inward barium currents arising from Ca_v_2.3 channels in stably transfected HEK-293 cells. Perfusing cells with 500 µM l-cystine solution blocked currents relative to the baseline. Red traces are recorded at − 10 mV test potential (inset: I–V protocol). **b** Normalized Ca_V_2.3 current densities at − 10 mV are shown for 7 cells. Perfusing cells with 500 µM l-cystine blocked currents significantly (paired two-tailed *t*-test, *P* < 0.0001). All currents are normalized to the maximal current density in baseline conditions. **c** Current density from 7 cells are drawn against test potentials in the presence (red) and absence (black) of 500 µM l-cystine. A significant decrease was observed upon perfusion at − 10 mV to 5 mV test potentials (two-way ANOVA *P* = 0.02 with Sidak multiple comparisons). **d** Normalized conductance of Ca_V_2.3 channels across test potentials are derived and sketched to compare the activation kinetics of Ca_v_2.3 channels with and without 500 µM l-cystine, where no difference was observed except at − 35 mV (two-way ANOVA P = 0.18 with Sidak multiple comparisons). **e** We next analyzed 10–90% rise time from current waveforms at different potentials. Although 10–90% rise (ms) for 500 µM l-cystine was trending lower than the baseline, the difference was not statistically significant. **f** Fast and slow time constants of activation were derived by fitting two-exponential function to current waveforms. **g** Fast and slow time constants of inactivation were derived by fitting two-exponential curves to current waveforms

In order to validate if l-cysteine manipulates currents arising from Ca_v_2.3 channels by oxidation mechanisms, we perfused Ca_v_2.3-transfected HEK-293 cells with its oxidized analog l-cystine at 500 µM (Fig. 3). L-cystine consists of two cysteine molecules bridged by an oxidized thiol group. Figure 3a depicts current traces for the baseline and perfusion. Red traces are recorded at − 10 mV test potential. Interestingly, we found that 500 µM l-cystine did not increase Ca_V_2.3 currents, and instead it partially blocked the currents. L-cystine inhibited peak amplitudes of Ca_V_2.3 currents by 50% in an apparently irreversible manner, suggesting a possible chemical nature of its mechanism (Fig. 3a, b). Figure 3c shows I–V curve for the baseline (black circles) and perfusion (red squares): while 500 µM l-cystine helps currents reach apex at lower membrane potentials, it also partially blocks the currents. Similar to l-cysteine, activation curve of the Ca_V_2.3 channels reveals that l-cystine shifted V_50_ of activation by − 10 mV (Fig. 3d). Although 10–90% rise time was trending lower after perfusing l-cystine, it was not significantly different (Fig. 3e). We also fitted and analyzed activation (Fig. 3f) and inactivation curves (Fig. 3g) for each cell and averaged among the test potentials to obtain fast and slow time constants; no significant difference was observed.

### Chelating agents increase Ca_V_2.3 currents

**Fig. 4:**
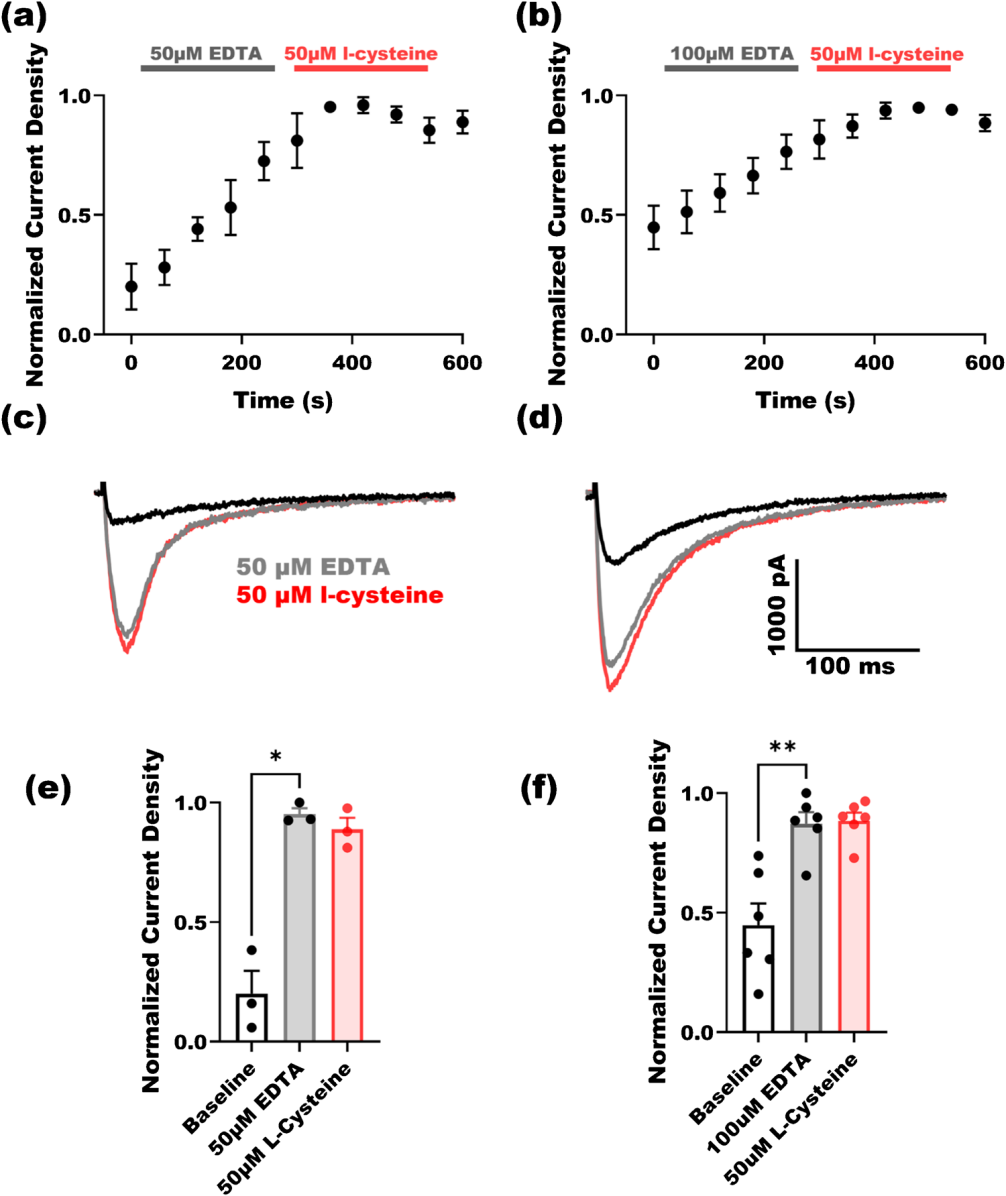
The effect of metal-chelating agent EDTA on recombinant Ca_V_2.3 currents. **a** At 50 µM, EDTA significantly increased inward currents in 3 cells (paired two-tailed *t*-test, *P* = 0.02), replacing EDTA with 50 µM l-cysteine in the bath did not cause an additional increase in currents in the same cells. **b** At 100 µM, EDTA significantly increased currents in 6 cells (paired two-tailed *t*-test, *P* = 0.003), replacing EDTA with 50 µM l-cysteine in the bath did not cause a further increase of currents in the same cells. **c**, **d** Traces of inward Ca_V_2.3 currents arising at − 10 mV test potential for 50 µM (**c**) and 100 µM (**d**) EDTA followed by 50 µM l-cysteine solution perfusion. **e**, **f** Perfusing 3 cells with 50 µM EDTA (**e**) and 6 cells with 100 µM EDTA (**f**) significantly increased the inward current and diminished any further l-cysteine effects. All currents are normalized to the maximal current density achieved with perfusion of EDTA

We previously demonstrated that chelating mechanisms play a role in enhancing T-type calcium currents in sensory neurons [8]. In order to examine whether chelating mechanisms may be contributing to current manipulation arising from recombinant Ca_V_2.3 channels, we perfused cells with a metal-chelating agent EDTA (Fig. 4). As expected, perfusing cells with EDTA at either 50 or 100 µM concentrations significantly increased currents in HEK-293 cells (Fig. 4a, b); however replacing EDTA in the bath with 50 µM l-cysteine did not increase the current any further (Fig. 4c, d). The data presented in Fig. 4 suggest that introducing a chelating agent to Ca_v_2.3 channels augments their currents similar to l-cysteine. Furthermore, we conclude that l-cysteine manipulates Ca_V_2.3 currents most likely through chelating constitutively bound trace metals rather than oxidizing mechanisms (Fig. 4e, f).

### In vivo pain studies show that Ca_v_2.3-deficient mice show hyperalgesia and different responses to injections of l-cysteine

**Fig. 5:**
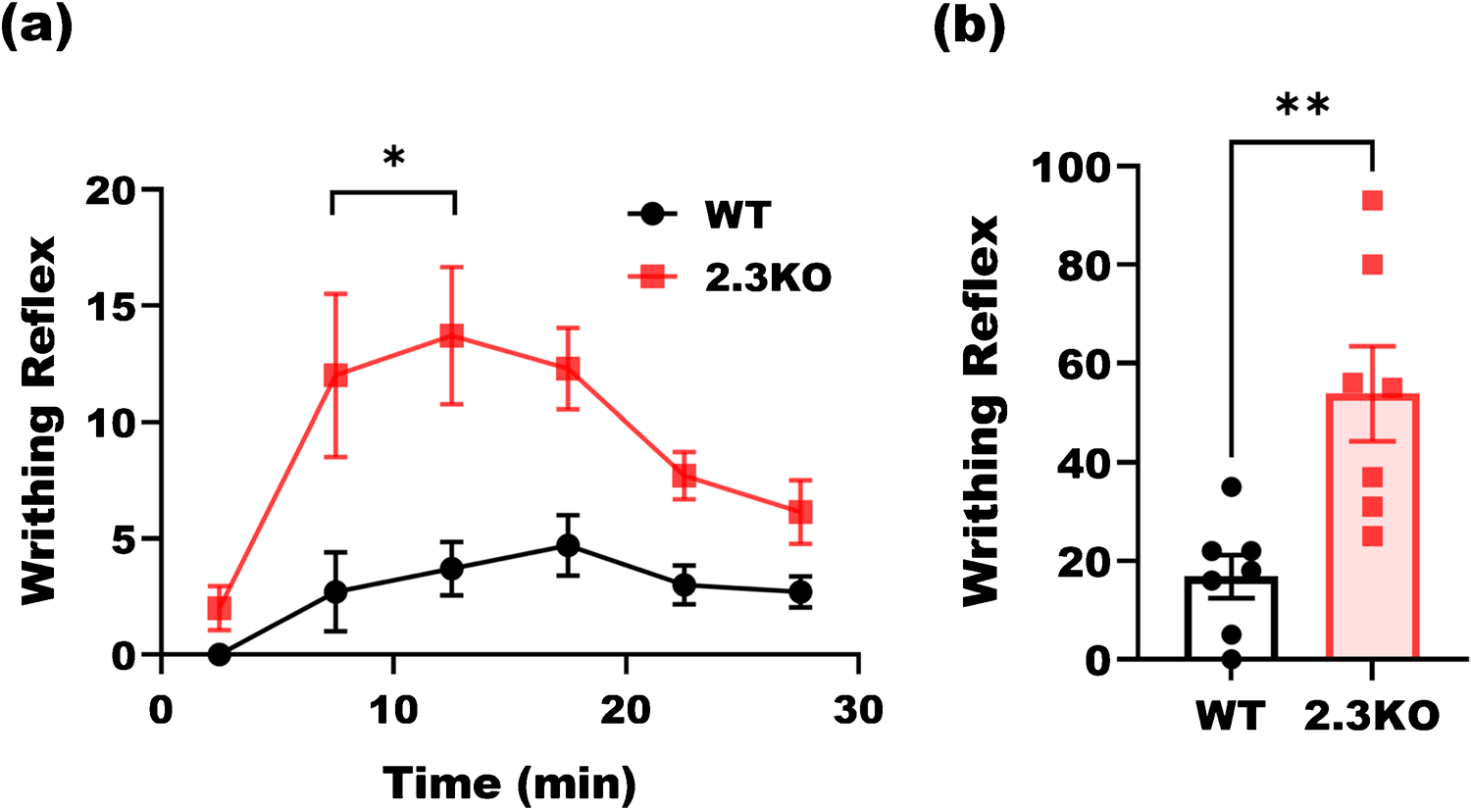
Role of Ca_v_2.3 channels in inflammatory visceral nociception. **a** Time course of pain reflex counts in acetic acid pain model in WT and Ca_V_2.3 KO male mice with 5-min intervals (two-way ANOVA *P* = 0.005 with Sidak multiple comparisons). **b** Total cumulative writhing reflex counts of 7 male WT and 2.3 KO mice (two-tailed *t*-test, *P* = 0.004)

**Fig. 6:**
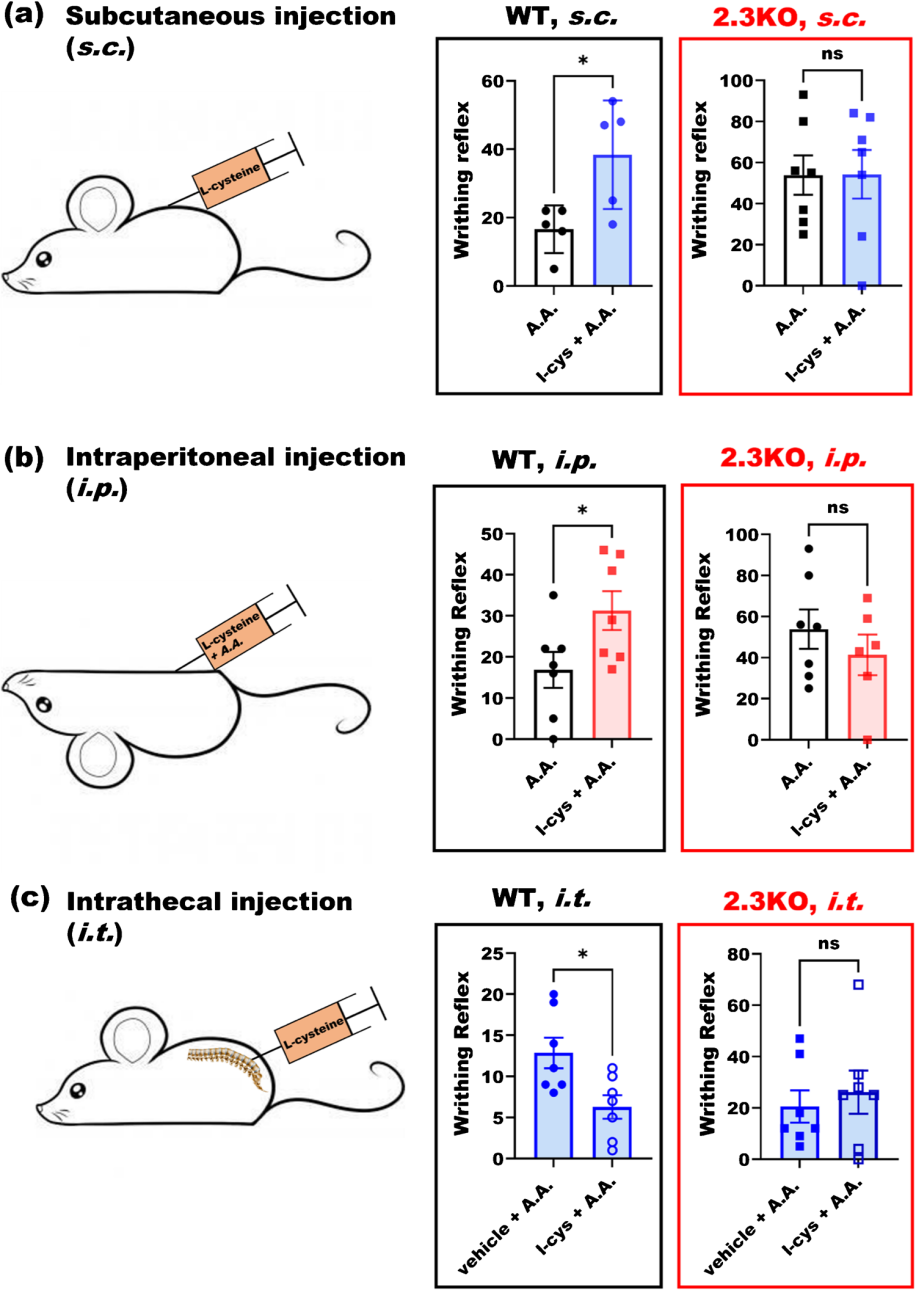
Different roles of Ca_v_2.3 channels in the periphery and lumbar spinal cord in inflammatory visceral nociception. **a** L-cysteine was injected s.c. to 5 WT and 7 KO male mice at 20 min prior to pain behavior study. L-cysteine significantly increased pain reflex counts (paired two-tailed *t*-test, *P* = 0.02) in WT littermates whilst its effect was abolished in KO subjects. **b** L-cysteine solution in 0.6% acetic acid was injected i.p. to 7 WT and KO male mice and pain reflexes were counted for 30 min. L-cysteine significantly increased pain reflex counts (paired two-tailed *t*-test, *P* = 0.04) in WT littermates, the effect was abolished in 2.3 KO subjects. **c** When l-cysteine was administered under isoflurane anesthesia to the lumbar spinal cord intrathecally 20 min prior to pain behavior study, it diminished hyperalgesia in WT male mice (paired two-tailed *t*-test, *P* = 0.03), whilst its effect was abolished in KO subjects

In order to investigate a possible role of Ca_v_2.3 channels in visceral pain processing, we compared pain behavior of wildtype (WT) and Ca_V_2.3 null (2.3 KO) littermates. We used pain model consisting of i.p*.* injections of 300 µl 0.6% acetic acid (A.A.) in saline and recorded the writhing reflexes of the subjects for 30 min [3]. Figure 5a shows the time course of pain behavior in male WT (black circles) and 2.3 KO mice (red squares). The writhing responses were greater in mice lacking Ca_v_2.3 channels at each time point up to fourfold (Fig. 5b), showing a significant role of these channels in this pain model for the first time. Since our patch-clamp data showed that l-cysteine has strong interactions with Ca_v_2.3 channels (see Figs. 1, 2 and 3), we proposed that it may be able to manipulate the pain behavior of WT subjects differently than in KO mice. Figure 6a compares the effect of systemic subcutaneous (s.c.) administration of l-cysteine (see the “Materials and methods” section) in WT and 2.3 KO mice. While the hyperalgesic effect in WT subjects was significant, it was abolished in the 2.3 KO mice. In Fig. 6b, the experiment was repeated by administering a very small dose of l-cysteine peripherally using i.p. injections together with injections of A.A. Similar to s.c. route, we found that l-cysteine administered i.p. increased pain reflex in WT littermates but not in 2.3 KO mice. Figure 6a and 6b together suggest that not only Ca_v_2.3 channels are involved in nociception, but also augmenting R-type calcium currents systemically or peripherally will increase pain sensitization. Next, we injected under anesthesia WT and 2.3 KO subjects intrathecally (i.t.) in the lower lumbar spinal cord with l-cysteine prior to the conducting visceral pain testing (Fig. 6c). Interestingly, in contrast to systemic and peripheral injections, we found that central administration of l-cysteine using i.t. route reduced the pain response in the WT subjects while the effect was abolished in Ca_v_2.3-deficient mice. Hence, our data demonstrate opposing roles of Ca_v_2.3 channels in the periphery and spinal cord in visceral pain transmission demonstrated through injections of l-cysteine. Interestingly, we noted that baseline writhing responses upon i.t. injections of l-cysteine were decreased in both WT and mutant cohorts when compared to baseline responses in our experiments with i.p. and s.c. injections. This may be due to use of relatively brief isoflurane anesthesia that is necessary for i.t. injections.

## Discussion

Herein, we first demonstrated that endogenous amino acid l-cysteine facilitates activation of Ca_V_2.3 channels and consequently enhances its current density likely by a metal-chelating mechanism. Further, we have demonstrated the opposing effects of intrathecally and systemically administered l-cysteine modulation of Ca_v_2.3 channels in A.A.-induced abdominal inflammatory pain model. We found that l-cysteine administrated i.t. partially blocks pain transmission in the spinal cord of WT littermates, while tis effect was abolished in Ca_v_2.3 KO mice. Meanwhile, s.c. and i.p. administration of l-cysteine induced pain sensitization in WT subjects, but not in Ca_v_2.3 KO littermates.

### Ca_v_2.3 currents are augmented with l-cysteine

Previous studies have investigated the interaction between l-cysteine and Ca_V_3.2 isoform of T-type calcium channels in the dorsal root ganglion (DRG) neurons [7–9]. It was shown that similar to this study, l-cysteine acts via chelating trace metals and increases T-current activation kinetics, and consequently increases subthreshold excitability. Knowing that R-type and T-type calcium channels have dynamic properties and structural similarities, we investigated the interactions between R-type calcium channels and endogenous l-cysteine. We hypothesized that l-cysteine may have the similar effect on these channels. Thus, using Ca_v_2.3-transfected HEK-293 cells, we recorded the inward currents using whole-cell patch-clamp recording. As expected, the results for both 50 and 500 µM L-cysteine showed increased current densities and facilitation of Ca_V_2.3 channel gating as demonstrated by a leftward shift in current activation curves.

### L-cystine partially blocks Ca_V_2.3 currents

Looking at the amino acid construct of the outer membrane segment of the Ca_v_2.3 channel, there are a few sites that resemble the cysteine residue of T-type calcium channels [10, 19]. Thus, we designed experiments to introduce cystine directly to the recording bath and study its effect on recombinant Ca_V_2.3 currents. In contrast to l-cysteine, we found that l-cystine partially blocked Ca_v_2.3 currents. Looking at the opposite results and what we observed with l-cysteine, we concluded that the mechanism of l-cysteine action is not oxidizing the thiol group on cysteine residues of Ca_V_2.3 channels.

### L-cysteine acts on Ca_V_2.3 channels likely through chelating the trace metals

Trace metals are highly regulated in the nervous system and serve as neuronal excitability co-factors [4, 6]. The abundance of certain trace metals (Zn^2+^ and Cu^2+^) has been reported in neurological disorders namely epilepsy, stroke, Alzheimer’s disease, and Parkinson’s [1, 4]. In contrast to mineral divalent ions, trace metals have extremely low concentrations in the nervous system (70–150 µM for Zn^2+^ and Cu^2+^) where a family of intracellular proteins, called metalloproteins, regulate them [6, 21]. While the vast body of trace metals are bound to these proteins, the free ion (chelatable) concentrations can reach up to 30 µM for Zn^2+^ and 1.7 µM for Cu^2+^. These “chelatable” free trace metal bodies can be found in the synaptic clefts in the cortex, limbic system, and basal ganglia [1]. In order to investigate the role of l-cysteine in chelating trace metals in the recording environment, we perfused cells with a known chelating agent EDTA and then replaced the bath solution with 50 µM l-cysteine. While chelating agent EDTA increased the Ca_V_2.3 current upon perfusion, subsequent l-cysteine perfusion effect was effectively occluded. Each experiment resulted in a finite current increase; however, once l-cysteine was introduced to the bath, a significant current increase was observed. Taken together, these results shed light on the action mechanism of l-cysteine and strongly suggest the metal-chelating properties of l-cysteine are the cause of current manipulation in Ca_v_2.3 channels.

### L-cysteine sensitizes visceral pain in WT mice

Although functional role of Ca_V_2.3 channels has been investigated in the previous studies [12, 13, 17], their thorough physiological properties remain elusive. The Ca_v_2.3 calcium channels are widely distributed throughout the thalamus (GABAergic reticular nucleus), cortex, and the spinal cord [22]. The distribution of Ca_v_2.3 channels in the main pain processing pathway, from dorsal horn spinal cord to the cortex, made us curious on their role in an abdominal inflammatory pain model. Somewhat surprisingly, our results show that Ca_v_2.3 KO mice exhibited more prominent visceral pain responses when compared to the WT littermates. The lower pain threshold in the global 2.3 KO subjects could be the result of removal of Ca_V_2.3 channels in the brain, spinal cord, and/or the periphery of null mice. Moreover, Ca_V_2.3 channels could serve as a novel analgesic target, thus we were interested to see if regulating the systemic level of l-cysteine would have any major effects on blocking pain in transgenic mice. We observed that systemic application of l-cysteine 20 min prior to the pain behavior assay or peripheral applications at the same time with A.A. increases pain responses in WT subjects significantly; however, the effect is largely diminished in KO subjects. These results together demonstrate the specificity of l-cysteine action to Ca_V_2.3 channels. However, dissecting their pharmacological target (central vs. periphery) requires further experiments. Our results are also not sufficient to exclude the possibility of the effect of l-cysteine on signaling pathways on other voltage-gated calcium channels, and Ca_V_3.2 isoform of T-type channels in particular [7–9, 14, 20]. Since both Ca_V_2.3 and Ca_V_3.2 channels are expressed in peripheral and central pain pathways, we conclude that the effects of L-cysteine on pain processing in vivo are likely mediated by both of these channels in concert. In addition, our results strongly suggest that Ca_V_2.3 channels are not saturated with endogenous levels of chelators of trace metals in vivo, since we were still able to modulate nociception with exogenous application of L-cysteine in WT mice.

### L-cysteine partially blocks pain transmission in the spinal cord

In order to distinguish the role of Ca_V_2.3 channels in the spinal cord, we injected WT and KO littermates with l-cysteine i.t. 20 min prior to the pain behavior task. We found that WT subjects injected with l-cysteine i.t. had less prominent visceral pain reflexes compared to vehicle (saline). In contrast, the effect of i.t. injections of l-cysteine in the KO subjects was subliminal and there was no significant difference between drug and the vehicle, which again demonstrates the specificity of l-cysteine action on Ca_v_2.3 channels in the spinal cord. Although these results prove that spinally expressed Ca_v_2.3 channels are manipulated upon exposure to l-cysteine, further experiments are required to clarify the plausible mechanisms of interactions of l-cysteine with Ca_V_2.3 channels in the dorsal horn of the spinal cord, a main pain processing region.

## Conclusion

Herein, we have demonstrated the crucial role of Ca_V_2.3 channels in visceral pain transduction, and shown the acting mechanism of an endogenous chelating agent, l-cysteine, through chelating trace metals in the spinal cord to block pain transmission. In contrast, systemic and peripheral administration of l-cysteine augmented abdominal writing responses suggesting different roles for peripheral and central Ca_V_2.3 channels in visceral pain processing.
